# How Brazilian Schoolchildren Identify, Classify, and Label Foods and Beverages—A Card Sorting Methodology

**DOI:** 10.3390/ijerph20021296

**Published:** 2023-01-11

**Authors:** Luciana Jeremias Pereira, Clarice Perucchi Lopes, Mayara Lopes Martins, Patrícia de Fragas Hinnig, Patricia Faria Di Pietro, Pedro Henrique de Moura Araujo, Dalton Francisco de Andrade, Maria Alice Altenburg De Assis, Francilene Gracieli Kunradi Vieira

**Affiliations:** 1 Post-Graduation Program in Nutrition, Health Sciences Center, Federal University of Santa Catarina, Florianópolis 88040-900, Brazil; 2 Doctoral School of Nutrition and Food Sciences, Institute of Nutrition, University of Debrecen, H-4002 Debrecen, Hungary; 3 Informatics and Statistics Department, Technological Center, Federal University of Santa Catarina, Florianópolis 88040-900, Brazil

**Keywords:** food categorization, schoolchildren, online questionnaire, cluster analysis, semi-structured interviews

## Abstract

This study examined how Brazilian schoolchildren identified, classified, and labeled foods and beverages. Semi-structured interviews were conducted with 133 schoolchildren aged 7 to 10 years old from a public school located in southern Brazil in 2015. A set of cards with pictures of 32 food and beverage items from the web-based Food Intake and Physical Activity of Schoolchildren tool (Web-CAAFE) were used. Participants identified each item, formed groups for them based on similarity, and assigned labels for those groups. Student’s *t*-tests and analysis of variance (ANOVA) tests were used to verify the mean difference between the groups of items. K-means cluster analysis was applied to identify similar clusters. Schoolchildren made an average of 9.1 piles of foods and beverages that they thought were similar (±2.4) with 3.0 cards (±1.8) each. Five groups were identified: meats, snacks and pasta, sweets, milk and dairy products, and fruits and vegetables. The most frequently used nomenclature for labeling groups was taxonomic-professional (47.4%), followed by the specific food item name (16.4%), do not know/not sure (13.3%), and evaluative (health perception) (8.8%). The taxonomic-professional category could be applied to promote improvements in the identification process of food and beverage items by children in self-reported computerized dietary questionnaires.

## 1. Introduction

Dietary assessment is one of the most challenging activities in epidemiology, and when it comes to younger ages, these difficulties are increased. Children present peculiarities in dietary assessment such as their immaturity of cognitive skills to recall previous intake, in addition to insufficient literacy that can limit their knowledge of foods, recipes, and meals [1].

Children can become unmotivated during dietary self-report due to difficulties in identifying food items and meals in the applied instrument. Consequently, data accuracy can be significantly decreased in their dietary assessment [2]. There is no consensus on what age children reach the necessary cognitive maturity to accurately report dietary intake [3,4]. Even though a sufficient cognitive level for responding to 24 h dietary recall was reported to be at age 10, improvements have also been reported regarding children’s cognitive ability to provide accurate dietary reports at age 8, but only for the immediate past, i.e., within the previous 24 h [3,4,5,6].

Categorization is a cognitive process that enables us to organize objects into groups. Without these abilities, each item would be perceived as new, and it would not be possible to generalize its properties (for example, assume that because an apple is edible, other apples will be edible, too) [7]. Children can classify food and beverage items by various evaluative categories such as taxonomic (based on common nutritional properties or similarities among the items), health perception (healthy and unhealthy), and script categories (the moment when the food/meal is eaten). The way children categorize can be influenced by age, cognitive ability, and by their own life experiences throughout childhood [8,9,10,11,12,13]. Studies suggest that children’s food categorization abilities begin to improve at 2 years of age [7,11]. Savage and collaborators found that children aged 5 to 8 years old presented similarity with parents in creating categories of candy (93% similarity), which insinuates a common cognitive structural way to categorize food items [14].

Dietary assessment questionnaires must be developed based on the potential responses from a target audience. In Brazil, the web-based Food Intake and Physical Activity of Schoolchildren tool (Web-CAAFE) was developed based on previous paper-and-pencil questionnaires [15,16]. Usability tests confirmed the child’s ability to understand the instrument and the ease of self-completion in the school environment with minimal assistance [17]. Web-CAAFE was submitted to two validity studies using direct observation of school meals as the reference method. The first was conducted with schoolchildren aged 7 to 10 years old from the south of Brazil and showed 43% matches, 29% intrusions, and 28% omissions. The second was carried out with students aged 7 to 15 years old from the northeast and showed 81.4% matches, 7.1% intrusions, and 16.2% omissions [18,19]. The instrument also proved to be satisfactory regarding the assessment of reproducibility [20]. Web-CAAFE was used in Brazilian health monitoring studies, being applied to more than 10,000 students in recent years [21,22,23]. However, it is necessary to evaluate the instrument constantly to help reduce errors in the estimates provided by them. This process is essential to improve the practicality and accuracy of self-reporting dietary instruments. Knowing how children identify illustrations of food items in a questionnaire allows analyzing whether the instrument was well designed for the target audience. Thus, this study aimed to verify how children aged 7 to 10 years old identify, classify, and label foods and beverages from Web-CAAFE. The categorization may be used to facilitate food and beverage items search in a computerized dietary recall for children [8,9,10,24,25]. The results will also provide insights to improve the Web-CAAFE screens and to guide researchers to design nutrition intervention programs using the labels and classification of food groups according to the children’s answers.

## 2. Materials and Methods

### 2.1. Design and Study Population

The present investigation was a methodological study to evaluate the cognitive abilities of children aged 7 to 10 years old to identify, label, and categorize food and beverage items illustrated in the Web-CAAFE questionnaire.

Schoolchildren aged 7 to 10 years old were recruited from a public school during August and September of 2015 in Florianópolis, a capital city in southern Brazil. The chosen institution was selected intentionally because it was not included in previous studies of usability, validation, reproducibility, and monitoring of children’s health using the same instrument, the Web-CAAFE questionnaire [17,18,19,20,22,26].

Convenience sampling was conducted within randomly selected children. Inclusion criteria were schoolchildren enrolled in the 2nd to 5th grades who were present at school on the day of data collection; had written consent from parents or guardians; gave written consent themselves; and were absent of any physical or mental problems reported by teachers that could prevent participation. For the present study, a total of 278 children were invited to participate. A total of 165 parents or tutors had written an initial consent for the children’s participation. A total of 24 children were recruited for a pretest of the study script, while 8 were excluded due to absence (n = 7) and inability to complete the task (n = 1). The final sample was comprised of 133 children and all subjects gave personal consent to participate.

### 2.2. Sample Characterization

Demographic data were provided by the school. Age was categorized by 7 to 8 and 9 to 10 years old. Weight and height were measured according to standardized procedures [27]. Weight was measured on a portable digital scale—Marte^®^ PP 180 model (maximum capacity of 180 kg and precision of 100 g). For height, the AlturExata^®^ stadiometer with a precision of one millimeter (mm) was used. Body mass index (BMI) was calculated and converted into age- and gender-specific z-scores according to the World Health Organization [28]. Weight status was categorized as non-overweight (under and normal weight children; z-score < +1.0) or overweight (including obese children, z-score ≥ +1.0).

### 2.3. Study Setting

A card sort methodology similar to previous studies was applied [8,9,10,13]. Semi-structured individual interviews were conducted with each participant by trained interviewers. The interview followed a pretested script used in a previous pilot study. The task was conducted in two parts: (1) the identification of foods and beverages and (2) the categorization of these items into groups, followed by their labeling.

The used web-based and previous-day recall questionnaire, Web-CAAFE, is a self-reported Brazilian instrument which was designed to monitor weight status, food consumption, physical activity, sedentary behaviors, satisfaction with school meals, and participation and satisfaction with physical education classes in schoolchildren enrolled in the 2nd to 5th grades [17,18,20,29].

The food consumption section is divided into six structured eating events (breakfast, mid-morning snack, lunch, mid-afternoon snack, dinner, and evening snack), and each one is illustrated by 32 food and beverage items: rice, vegetables, green leaves, vegetable soup, beans (cooked), manioc flour, maize/potatoes, pasta, instant pasta, French fries, beef/poultry, sausages, eggs (fried, boiled, or omelet), fish/seafood, fruits (traditional Brazilian fruits such as bananas and oranges), bread/biscuits, cheese bread, cream cookie, breakfast cereal, porridge, cheese, coffee with milk, milk, yogurt, chocolate milk, fruit juices, sodas, sweets (such as candies, chocolate bars, ice cream, and cakes with icing), chips, pizza/hamburger/hot-dog, nuggets, and cakes (Figure S1). More details on the development, validation, and reliability of the instrument are described in other studies [17,18,19,20]. The demo version of the questionnaire, including English subtitles, is available at http://caafe.ufsc.br/portal/10/detalhes, accessed on 14 August 2020.

The food and beverage Items selection took into account their frequency of intake in this age group as reported previously by 180 schoolchildren in 7-day food diaries, the food presented in school cafeteria menus [17], as well as the food recommended by the Dietary Guidelines for the Brazilian Population (DGBP) [30,31].

#### 2.3.1. Identification of Food and Beverage Items (First Part)

For the first part, trained investigators presented a set of cards (6 cm × 6 cm) randomly sorted containing colored images of 32 food and beverage items presented in the Web-CAAFE questionnaire. Next, the children were individually asked to identify and name each item in rounds. An option for the answer “I don’t know” was suggested in case of unknown items.

#### 2.3.2. Categorization of Items Followed by Labelling (Second Part)

Subsequently, children were told to sort the cards into piles according to similarities. In order to facilitate comprehension, interviewers provided examples using images of different foods or beverages that were not included in the Web-CAAFE screens. Participants were advised to make as many piles as they wanted, and that there was no right or wrong way to do so. If they had doubts about any item, an “I am not sure” pile was permitted. In addition, the investigators explained that it was possible to change cards from piles as much as they wanted during the interview. After choosing piles for all 32 cards, the children were asked if they wanted to move any item from the “I don’t know” or “I am not sure” piles to other ones. At the end, children were asked individually to label each pile and justify the chosen name. The full interview was recorded.

### 2.4. Data Processing

Firstly, the names given by children for each food and beverage item were standardized. For instance, participants gave several names to the item that visually represented vegetables, such as veggies, vegetables, salad greens, and salad. Thus, in order to organize and maintain the original meaning, the final name was standardized by the investigators to “vegetables/salad”.

Posteriorly, the second part of data processing was conducted by two different nutritionists in two stages. This strategy was used in order to guarantee uniformity and consistency among the labels created by the schoolchildren for each pile [8,9,10,13].

Therefore, the first-level labeling considered the original name given by participants for each pile (raw data). For the second-level labeling, first-level names were converted into similar labels maintaining the basic integrity of the initial name. For example, when the first-level labels were ‘foods made from fruits’ and ‘things you can make with fruit’, nutritionists standardized it to the ‘made from fruit’ label (second-level label).

At the third-level labeling, second-level names were categorized into nine conceptual categories that reflected children’s cognitive development: (1) evaluative (preferences), (2) specific food item name, (3) food characteristics, (4) script scheme, (5) food preparation, (6) thematic (combination), (7) evaluative (health perception), (8) taxonomic-professional, and (9) nutrient composition (Table 1) [8,9,10,13,32].

### 2.5. Statistical Analysis

Absolute and relative frequencies were used to characterize the sample and to describe the frequencies of identification (correct, incorrect, and unknown items) of 32 food and beverage items accordingly labeled in the Web-CAAFE screens. Mean, standard deviation (SD), and confidence interval 95% (CI95%) were calculated and Student’s t-tests and analysis of variance (i.e., ANOVA) tests were used to verify the mean difference between the formed piles according to the characteristics of the sample. A 32 × 32 proximity matrix was created using Microsoft Excel^®^ [33] and R version 3.3.1 [34] software to reflect the relationship among items in the piles created by the schoolchildren. A combination of hierarchical and non-hierarchical clustering method was performed to identify the number of clusters created from the 32 food and beverage items by schoolchildren. The hierarchical procedure was carried out based on Euclidean distances and was performed to identify and compare several possibilities of cluster solutions. After that, the non-hierarchical k-means clustering procedure was performed to better fit the preliminary hierarchical solution. The names of the clusters were designated according to the frequency of labels given by the schoolchildren. Cross-tabulation method was performed to describe the absolute and relative frequencies of food and beverage items in the conceptual categories. The absolute and relative frequencies of conceptual categories were also described separately according to age.

## 3. Results

Table 2 shows sociodemographic and weight status characteristics of the sample according to the number of piles. A total of 133 schoolchildren participated in this study, most of whom were girls (63.4%, n = 84) aged from 9 to 10 years old (57.1%, n = 76). According to school grades, 36.1% (n = 42) were enrolled in the 3rd grade. Only 15.8% (n = 21) were overweight (including obesity). Participants created an average of 9.1 (±2.4) piles of items with 3.0 cards (±1.8) each. The number of formed piles did not show significant association with any sociodemographic characteristic (Table 2).

All participants identified and named the cards containing “eggs”, “cheese”, “milk”, and “cream cookie” as they were originally nominated in the Web-CAAFE questionnaire. The “nuggets” were identified correctly by 45.9% (n = 61) of the total sample, while 28.6% (n = 38) were unable to name it. The “pasta” was the second item with the lowest frequency of identification (57.8%, n = 77). More than 90.0% of the schoolchildren properly identified the following items: “rice”, “vegetable soup”, “beans (cooked)”, “instant pasta”, “French fries”, “breakfast cereal”, “fruits”, “bread/biscuit”, “cake”, “porridge”, “coffee with milk”, “yogurt”, “chocolate milk”, “fruit juices”, “sodas”, “sweets”, and “chips” (Table 3).

**Figure 1:**
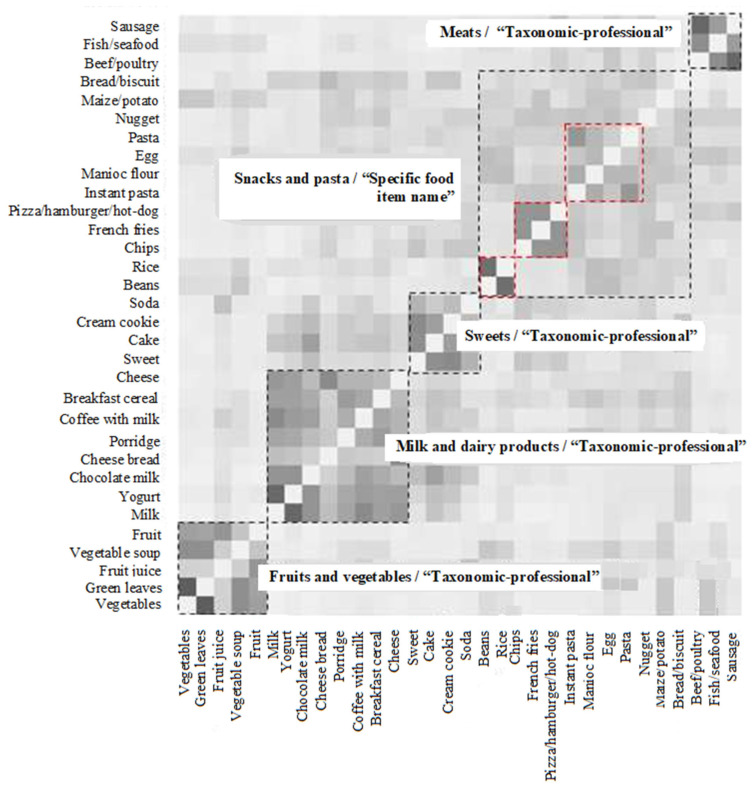
Clusters derived by cluster analysis.

Overall, five clusters were derived from the categorizations performed by the schoolchildren. Figure 1 shows a dendrogram considering these five clusters with the most used labels by schoolchildren: (1) “fruits and vegetables”, (2) “milk and dairy products”, (3) “sweets”, (4) “snacks and pasta”, and (5) “meats”. It was possible to identify three sub-clusters overlapping the “snack and pasta” cluster whose food items were (a) pasta, eggs, manioc flour, and instant pasta; (b) pizza/hamburger/hot-dog; and (c) rice and beans. The clusters 3 (“sweets”) and 5 (“meats”) showed the lowest intra-group variations (15625.00 and 15512.00, respectively) and cluster 4 (“snacks and pasta”) presented the highest variation (54042.83) (Table S1).

Table 4 shows the frequency of the 32 food and beverage items at the third-level labeling. A total of 278 names for piles were provided by schoolchildren at the first-level labelling, and posteriorly coded into 36 second-level labels. Later, these 36 nomenclatures were classified into conceptual categories. Most food and beverage items were classified in the “taxonomic-professional” category (47.4%, n = 1998), followed by the “specific food item name” (16.4%, n = 657), “don’t know” or “not sure” categories (13.3%, n = 584), and “evaluative (health perception)” (8.8%, n = 372) (Table 4). Similar results were observed in the frequencies of conceptual categories according to age (Table S2).

The five food items with the highest frequency of classification in the “taxonomic-professional” category were “milk” (86.5%, n = 115), “yogurt” (85.6%, n = 113), “beef/poultry” (74.4%, n = 99), “sausage” (73.3%, n = 96), and “chocolate milk” (68.7%, n = 90) (Table 4). The five food items with the highest frequency of classification in the “specific food item name” category were “cheese bread” (35.9%, n = 47), “cheese” (35.6%, n = 47), “bread/biscuits” (32.0%, n = 41), “beans” (29.8%, n = 39), and “French fries” (27.8%, n = 33) (Table 4). The “nuggets” and “manioc flour” items had the highest frequencies in the “don’t know” or “not sure” categories with 33.1% (n = 43) and 32.8% (n = 43), respectively (Table 4). The five food items with the highest frequency of classification in the “evaluative (healthy perception)” category were “vegetables” (30.5%, n = 40), “green leaves” (28.8%, n = 38), “fruits” (22.9%, n = 30), “vegetables soup” (18.1%, n = 24), and “pizza/hamburger/hot-dog” (16.0%, n = 21) (Table 4).

## 4. Discussion

The present study described how Brazilian schoolchildren aged 7 to 10 years old identified, classified, and labeled food and beverage items based on the Web-CAAFE questionnaire. This study contributes to the area of nutritional epidemiology, specifically to the development and improvement of food consumption instruments to this age group. The observed results showed that most of the items were identified similarly as their original names in the instrument, which indicates that the images used in the questionnaire were interpreted correctly by the target population. A proper comprehension of the applied instrument can promote better practicality in filling it out, and more accuracy in the collected data [35].

In contrast, schoolchildren had greater difficulty in identifying the “nuggets” and “pasta” items. This cognitive task required children to recognize food, food preparations, and cooking methods in detail and that may not have been compatible with the perceptual and conceptual capacities of children who have not reached the stage of abstract reasoning (approximately 10–11 years) [5,36,37]. Later studies have already improved the methodology for application of such a questionnaire by trained researchers [20,21,23].

Five clusters were derived from the grouping of 32 Web-CAAFE items. Other studies have also assessed food item categorization by children. An American study with 146 children aged 8 to 13 years old observed 8 clusters with 62 food and beverage items through the Robinson matrix procedure [10]. Later, the same research group found out that children categorized 71 grain foods into 6 clusters [9] and 48 mixed foods (i.e., lasagna, peanut butter and jelly sandwich, pizza, etc.) into 9 clusters [8]. Recently, Savage et al. [14] reported that children aged 5 to 8 years old created 8 clusters of candy products and most of them (n = 7) were similar to the ones formed by their parents (93% similarity).

The cluster consisting of beef/poultry, fish/seafood, and sausages items were mostly labelled as the “meat” group, a taxonomic-professional classification. This result corroborates with another study which demonstrated the same categorization for sausages, beef, chicken, shrimp, pork, and fish among American children aged 8 to 13 years old [8].

Likewise, taxonomic-professional categorization was used by most of the children to cluster the items of coffee with milk, milk, yogurt, cheese bread, cheese, breakfast cereal, and chocolate milk as the “milk and dairy products” group. In contrast to this finding, Beltran et al. [8] observed different clusters for similar items among 8–13-year-old children. For instance, milk was classified in the taxonomic-professional category as the “drinks” group, but yogurt and cheese were clustered as “healthy snacks” (evaluative (healthy perception) category), and ice cream and pudding/flan as “snacks” (script scheme category).

In the present study, the traditional Brazilian item “cheese bread” was categorized as “milk and dairy products”. Thus, schoolchildren did not consider the main ingredient of this item, the manioc starch (45% carbohydrates), which indicates that “cheese bread” may be mainly associated with the cheese component. In addition, the breakfast cereal was mostly clustered as the “milk and dairy products” group. This result can be explained by the image given in the questionnaire, since it is represented by a bowl filled with breakfast cereal and milk. Furthermore, schoolchildren may have associated both foods intentionally due to an external inductive inference (association of two food items consumed together or consumed in the same meal) [38].

The cluster “sweets” was chosen for the items of sweets, cream cookie, and sodas, whose items had a higher prevalence of taxonomic classification. A similar classification was also observed in a previous American study with 115 children aged 5 to 11 years old [39]. In addition, a recent study identified that children aged 5–8 years old were able to categorize 51 types of candy into 8 different clusters: “chocolate”, “candied nuts and dried fruit”, “hard candy”, “sugar candy”, “chewy candy”, “caramels”, “gum and mints”, and “sugar-free candy” [14].

Children created a cluster labeled “fruits and vegetables” that includes fruits, vegetables, green leaves, vegetable soup, and fruit juice items. This cluster was classified in the taxonomic-professional category. In the United States, 8–13-year-old children created two different clusters: “fruits” including apple sauce, banana, and fruit cocktail items and “vegetables/vegetable juice” with corn, tossed salad, mashed potatoes, and French fries items [8]. Moreover, our study demonstrated that Brazilian children classified the maize/potatoes into a “snack and pasta” cluster, indicating that they are able to distinguish between non-starchy and starchy vegetable, but do not categorize them in the same group.

The “snack and pasta” was the most heterogeneous cluster and perhaps this explains the fact that students label this cluster with the specific names of the foods instead of using some specific knowledge about professional grouping or health effects of food items. In a previous study, 8–13-year-old children created two similar clusters and classified them with same “specific food item name” category: rice and soups/noodles/pasta [8]. In addition, in the present study, it was possible to identify three sub-clusters overlapping the “snack and pasta” cluster whose food items were (a) pasta, eggs, manioc flour, and instant pasta; (b) pizza/hamburger/hot-dog, chips, and French fries; and (c) rice and beans. The fact that schoolchildren created a sub-cluster with the “rice” and “beans” items may suggest a perception about the complementary relationship between these items, since these are the basic foods of the traditional Brazilian diet and are consumed together [31].

Schoolchildren tended to classify most foods as taxonomic-professional, followed by the specific food item name, do not know or not sure, and evaluative (health perception) categories, indicating that they used a certain level of knowledge about professional groupings and health effects of food items, or simply reported the concrete characterization or name of the food and beverage item contained in the card. In previous studies, children also grouped most food items into taxonomic-professional categories, but also shared cultural knowledge when using script scheme and thematic categories to label their clusters [8,9,10,13]. In our study, only 4.2% (n = 161) of schoolchildren used the script scheme categories to label their piles. However, it was observed that script categories may not be the most effective when compared to the taxonomic-professional category for clustering food items [40].

Food items were also simultaneously classified into other categories. For instance, the “maize/potatoes” item was similarly distributed among the “taxonomic-professional”, “specific food item name”, and “don’t know” or “not sure” categories, indicating conceptual flexibility at the group level [41]. Beltran et al. [8,10] previously reported that children can identify the same food items into different categories depending on the context. In these studies, French fries and milk items were firstly classified in snack/script and drinks/taxonomic-professional categories in a card sort activity composed of single foods, respectively. Posteriorly, the same items were classified in junk food-unhealthy/evaluative and dairy/taxonomic-professional in a mixed dishes card sort activity, respectively. These findings indicate that the same food item can be listed in several food categories to facilitate its location in self-reported computerized dietary questionnaires. In addition, a study conducted by Nguyen [38] examined children’s tendency to use evaluative versus taxonomic categorization and showed that younger children (average age: 5 years) tended to use an evaluative category for foods more than the older ones (average age: 7 years), which suggests that taxonomic knowledge is available later compared to the evaluative knowledge.

This study contains several strengths and limitations. Among the strong points, the interviews were performed by trained researchers with semi-structured script based on previous methodologies [8,9,10,13,39] and a pretest was performed in a sub-sample of the studied population. Additionally, data processing was performed by two researchers. Among the limitations, the studied school and sample were obtained by convenience, which may limit some inferences in the present study. However, it should be noted that the school receives students from different regions of Florianópolis and neighboring cities, presenting different socioeconomic and cultural conditions.

## 5. Conclusions

This study confirms that children successfully identify most of the items from a Web-CAAFE questionnaire. The “nuggets” and “pasta” items had the lowest frequency of correct identification by the studied population. Web-CAAFE is a technology-based dietary assessment measure that must be frequently evaluated and updated to improve accuracy and reduce participant burden. Findings from the current study conducted in 2015 provided practical implications to the subsequent studies that used the Web-CAAFE tool. In these studies, the item “nuggets” was deleted from the screens of the tool and the protocol for training the researchers was standardized in order to provide better explanations about food items [20,21,23]. In addition, future studies should verify how the illustration of these specific foods can be improved considering the limited cognitive capacity of children to recognize certain food items. We also could infer that children have cognitive skills to group foods within the taxonomic-professional category, but interestingly, we found that children have a perception of health when food items such as vegetables, green leaves, fruits, vegetables soup, and pizza/hamburger/hot-dog had the highest frequency of classification in the evaluative (healthy perception) category, indicating that this new approach could be applied to promote improvements in schoolchildren questionnaires. These findings also have important implications for food and nutrition education and health promotion actions.

## Figures and Tables

**Table 1:** Conceptual categories applied in the third-level categorization.

Cognitive Level	Conceptual Categories	Examples
Individual characteristic	1. Evaluative: preferences	Like or do not like
Concrete characterization of the food	2. Specific food item name	Name of the picture
	3. Food characteristics	Colors, texture, taste, and shape
Requires some knowledge of a common culture	4. Script scheme	Scheme for a routine or event: lunch, snack, birthday, dinner, etc.
	5. Food preparation	Baked, cooked, frozen, packaged, etc.
	6. Thematic: combination	Food/ingredient groups that are associated with or have a complementary relationship (i.e., rice and cooked beans)
Requires some knowledge or perception of the health effects of food items	7. Evaluative: health perception	Good or bad, healthy or unhealthy
Requires knowledge of professional groupings	8. Taxonomic-professional	Based on common properties or similarities among the categories (beverages, grains, dairy, plant-related, farm group, etc.)
	9. Nutrient composition	Macro and micro nutrients (proteins, fats, carbohydrates, and vitamins and minerals)

Adapted from Beltran et al. [8].

**Table 2:** Sociodemographic characteristics according to number of piles of 7–10-year-old schoolchildren, Florianópolis, Southern Brazil, 2015.

Characteristic	n	%	Piles (n)		
			Mean	SD	CI 95%
Sex *^,^§					
Girls	84	63.4	9.0	2.7	8.4–9.6
Boys	49	36.6	9.3	2.1	8.7–9.9
Total	133	100.0	9.1	2.4	8.7–9.5
Age (years) *^,^§					
7 to 8	57	42.9	9.0	2.6	8.3–9.7
9 to 10	76	57.1	9.2	2.3	8.7–9.3
School Grade †^,^§					
2°	32	24.1	8.8	3.2	7.6–9.9
3°	42	36.1	9.1	2.1	8.5–9.8
4°	33	24.8	9.3	2.4	8.5–10.1
5°	26	19.5	9.3	2.1	8.4–10.1
Weight status (BMI) ^a,^*^,^§					
Non-overweight	112	84.2	9.3	2.5	8.8–9.7
Overweight (including obesity)	21	15.8	8.3	2.4	7.3–9.3

n: absolute frequency; % relative frequency; CI 95%: confidence interval 95%; SD: standard deviation; * Student’s *t*-test; † ANOVA test; § significance level *p* > 0,05; ^a^ World Health Organization [28].

**Table 3:** Frequencies of identification of 32 food and beverage items from Web-CAAFE by 7–10-year-old schoolchildren, Florianópolis, Southern Brazil, 2015.

Food and Beverage Items from Web-CAAFE	Correctly Identified		Incorrectly Identified		Unknown Items	
	n	%	n	%	n	%
Rice	125	94.0	7	5.2	1	0.8
Vegetables	106	79.7	25	18.8	2	1.5
Green leaves	90	67.7	42	31.5	1	0.8
Vegetable soup	126	94.7	6	4.5	1	0.8
Beans (cooked)	130	97.7	3	2.3	0	0.0
Manioc flour	114	85.7	18	13.5	1	0.8
Pasta	77	57.8	53	39.9	3	2.3
Instant pasta	123	92.5	9	6.7	1	0.8
French fries	124	93.2	9	6.7	0	0.0
Beef/poultry	115	86.5	17	12.7	1	0.8
Eggs	133	100.0	0	0.0	0	0.0
Fish/seafood	80	60.2	53	39.8	0	0.0
Maize/potatoes	112	84.2	21	15.8	0	0.0
Sausages	100	75.2	32	24.0	1	0.8
Nuggets	61	45.9	34	25.5	38	28.6
Breakfast cereal	129	97.0	3	2.3	1	0.8
Fruits	123	92.5	10	7.5	0	0.0
Bread/biscuits	127	95.5	6	4.5	0	0.0
Cheese bread	114	85.7	19	14.3	0	0.0
Cake	123	92.5	10	7.5	0	0.0
Porridge	122	91.8	9	6.7	2	1.5
Cheese	133	100.0	0	0.0	0	0.0
Coffee with milk	123	92.5	10	7.5	0	0.0
Milk	133	100.0	0	0.0	0	0.0
Yogurt	131	98.5	2	1.5	0	0.0
Chocolate milk	126	94.7	7	5.3	0	0.0
Fruit juices	129	97.0	4	3.0	0	0.0
Cream cookie	133	100.0	0	0.0	0	0.0
Sodas	131	98.5	2	1.5	2	1.5
Sweets	124	93.3	9	6.7	0	0.0
Chips	126	94.7	6	4.5	1	0.8
Pizza/hamburger/hot-dog	95	71.4	37	27.8	1	0.8

n = absolute frequency; % = relative frequency.

**Table 4:** Frequencies of 32 food and beverage items from Web-CAAFE according to conceptual categories.

Food and Beverage Items	Evaluative: Preferences	Specific Food Item Name	Food Characteristic	Script Scheme	Food Preparation	Thematic: Combination	Evaluative: Health Perception	Taxonomic-Professional	Nutrient Composition	Don’t Know/ Not Sure
	n (%)	n (%)	n (%)	n (%)	n (%)	n (%)	n (%)	n (%)	n (%)	n (%)
Fruits (n = 131)	-	6 (4.6)	1 (0.8)	2 (1.5)	-	1 (0.8)	30 (22.9)	78 (59.5)	1 (0.8)	12 (9.2)
Vegetables (n = 131)	-	8 (6.1)	2 (1.5)	1 (0.8)	-	-	40 (30.5)	72 (55.0)	-	8 (6.1)
Green leaves (n = 132)	-	8 (6.1)	4 (3.0)	2 (1.5)	-	-	38 (28.8)	70 (53.1)	-	10 (7.6)
Vegetable soup (n = 133)	1 (0.8)	16 (12.0)	13 (9.8)	7 (5.3)	-	1 (0.8)	24 (18.1)	51 (38.6)	-	20 (15.0)
Fruit juices (n = 131)	-	17 (13.0)	3 (2.3)	5 (3.8)	-	2 (1.5)	15 (11.5)	79 (60.3)	1 (0.8)	9 (6.9)
Beans (cooked) (n = 131)	3 (2.3)	39 (29.8)	9 (6.9)	15 (11.5)	-	8 (6.1)	8 (6.1)	19 (14.5)	1 (0.8)	29 (22.1)
Rice (n = 132)	2 (1.5)	35 (26.5)	10 (7.6)	13 (9.9)	1 (0.8)	7 (5.3)	8 (6.1)	26 (19.7)	1 (0.8)	29 (22.0)
Manioc flour (n = 131)	4 (3.1)	30 (22.9)	13 (9.9)	8 (6.1)	1 (0.8)	5 (3.8)	2 (1.5)	24 (18.3)	1 (0.8)	43 (32.8)
Pasta (n = 131)	2 (1.5)	24 (18.3)	9 (6.9)	6 (4.6)	2 (1.5)	1 (0.8)	5 (3.8)	53 (40.5)	1 (0.8)	28 (21.4)
Eggs (n = 131)	2 (1.5)	24 (18.3)	14 (10.7)	12 (9.2)	2 (1.5)	2 (1.5)	8 (6.1)	30 (22.9)	-	37 (28.2)
Instant pasta (n = 133)	3 (2.3)	29 (22.0)	19 (14.4)	3 (2.3)	2 (1.5)	-	7 (5.3)	38 (28.8)	-	32 (24.2)
French fries (n = 133)	2 (1.5)	37 (27.8)	17 (12.8)	3 (2.3)	8 (6.0)	-	17 (12.8)	35 (26.3)	2 (1.5)	12 (9.0)
Maize/potatoes (n = 131)	-	35 (26.7)	9 (6.8)	5 (3.8)	1 (0.8)	2 (1.5)	10 (7.6)	35 (26.7)	1 (0.8)	33 (25.2)
Nuggets (n = 130)	-	22 (16.9)	12 (9.3)	5 (3.8)	4 (3.1)	-	10 (7.7)	33 (25.4)	1 (0.8)	43 (33.1)
Bread/biscuits (n = 128)	-	41 (32.0)	5 (3.9)	14 (10.9)	1 (0.8)	3 (2.3)	4 (3.1)	35 (27.3)	3 (2.3)	22 (17.2)
Chips (n = 131)	2 (1.5)	26 (1.5)	25 (19.1)	1 (0.8)	2 (1.5)	1 (0.8)	18 (13.7)	44 (33.6)	2 (1.5)	10 (7.6)
Pizza/Hamburger/hot-dog (n = 131)	2 (1.5)	22 (16.8)	16 (12.2)	4 (3.0)	5 (3.8)	-	21 (16.0)	46 (35.1)	2 (1.5)	13 (9.9)
Cream cookie (n = 131)	-	20 (15.3)	6 (4.6)	7 (5.3)	-	1 (0.8)	12 (9.2)	74 (56.5)	-	11 (8.4)
Cake (n = 131)	-	28 (21.4)	4 (3.0)	5 (3.8)	-	-	8 (6.1)	78 (59.5)	-	8 (6.1)
Soda (n = 132)	-	26 (19.7)	8 (6.1)	3 (2.3)	-	-	12 (9.1)	59 (44.7)	-	24 (18.2)
Sweets (n = 133)	-	15 (11.3)	2 (1.5)	2 (1.5)	-	-	20 (15.0)	87 (65.4)	1 (0.8)	6 (4.5)
Beef/poultry (n = 133)	-	7 (5.3)	7 (5.3)	5 (3.8)	1 (0.8)	-	5 (3.8)	99 (74.4)	1 (0.8)	8 (6.0)
Sausage (n = 131)	-	5 (3.8)	4 (3.0)	4 (3.0)	2 (1.5)	1 (0.8)	6 (4.6)	96 (73.3)	1 (0.8)	12 (9.2)
Fish/seafood (n = 132)	-	8 (6.1)	6 (4.5)	4 (3.0)	1 (0.8)	-	10 (7.6)	80 (60.6)	-	23 (17.4)
Coffee with milk (n = 132)	-	14 (10.6)	5 (3.8)	11 (8.3)	-	-	2 (1.5)	83 (62.9)	-	17 (12.9)
Porridge (n = 131)	-	18 (13.7)	11 (8.4)	4 (3.0)	-	-	6 (4.6)	62 (47.3)	-	30 (22.9)
Cheese (n = 133)	-	47 (35.6)	2 (1.5)	4 (3.0)	-	-	3 (2.3)	68 (51.5)	-	9 (6.8)
Cheese bread (n = 131)	2 (1.5)	47 (35.9)	7 (5.3)	3 (2.3)	1 (0.8)		8 (6.1)	47 (35.9)	1 (0.8)	15 (11.5)
Milk (n = 133)	-	7 (5.3)	1 (0.8)	4 (3.0)	-	-	2 (1.5)	115 (86.5)	1 (0.8)	3 (2.3)
Yogurt (n = 132)	-	3 (2.3)	1 (0.8)	3 (2.3)	-	2 (1.5)	4 (3.0)	113 (85.6)	1 (0.8)	5 (3.8)
Chocolate milk (n = 131)	-	26 (19.8)	5 (3.8)	1 (0.8)	-	-	4 (3.1)	90 (68.7)	1 (0.8)	4 (3.1)
Breakfast cereal (n = 131)	-	9 (6.9)	10 (7.6)	9 (6.9)	-	-	5 (3.8)	79 (60.3)	-	19 (14.5)
Total	24 (1.6)	657 (16.4)	260 (6.2)	161 (4.2)	34 (1.7)	37 (2.0)	372 (8.8)	1998 (47.4)	24 (1.0)	584 (13.3)

n = absolute frequency; % = relative frequency.

## Data Availability

The data presented in this study are available upon request from the corresponding author.

## Supplementary Materials

The following supporting information can be downloaded at: https://www.mdpi.com/article/10.3390/ijerph20021296/s1, Figure S1: Food and beverage items from Web-CAAFE questionnaire. Table S1. Intra-group variation values of the five clusters derived from the k-means non-hierarchical cluster analyses, Florianópolis, Southern Brazil, 2015. Table S2. Frequencies of conceptual categories by age of 7–10-year-old schoolchildren, Florianópolis, Southern Brazil, 2015.
