# Supplementary material for: How Brazilian Schoolchildren Identify, Classify, and Label Foods and Beverages—A Card Sorting Methodology
Source: Int J Environ Res Public Health. 2023 Jan 11;20(2):1296. doi: 10.3390/ijerph20021296 (PMC9859169; doi:10.3390/ijerph20021296)
Supplement: Supplementary file 1 [file ijerph-20-01296-s001.zip › ijerph-2085441-supplementary.pdf]

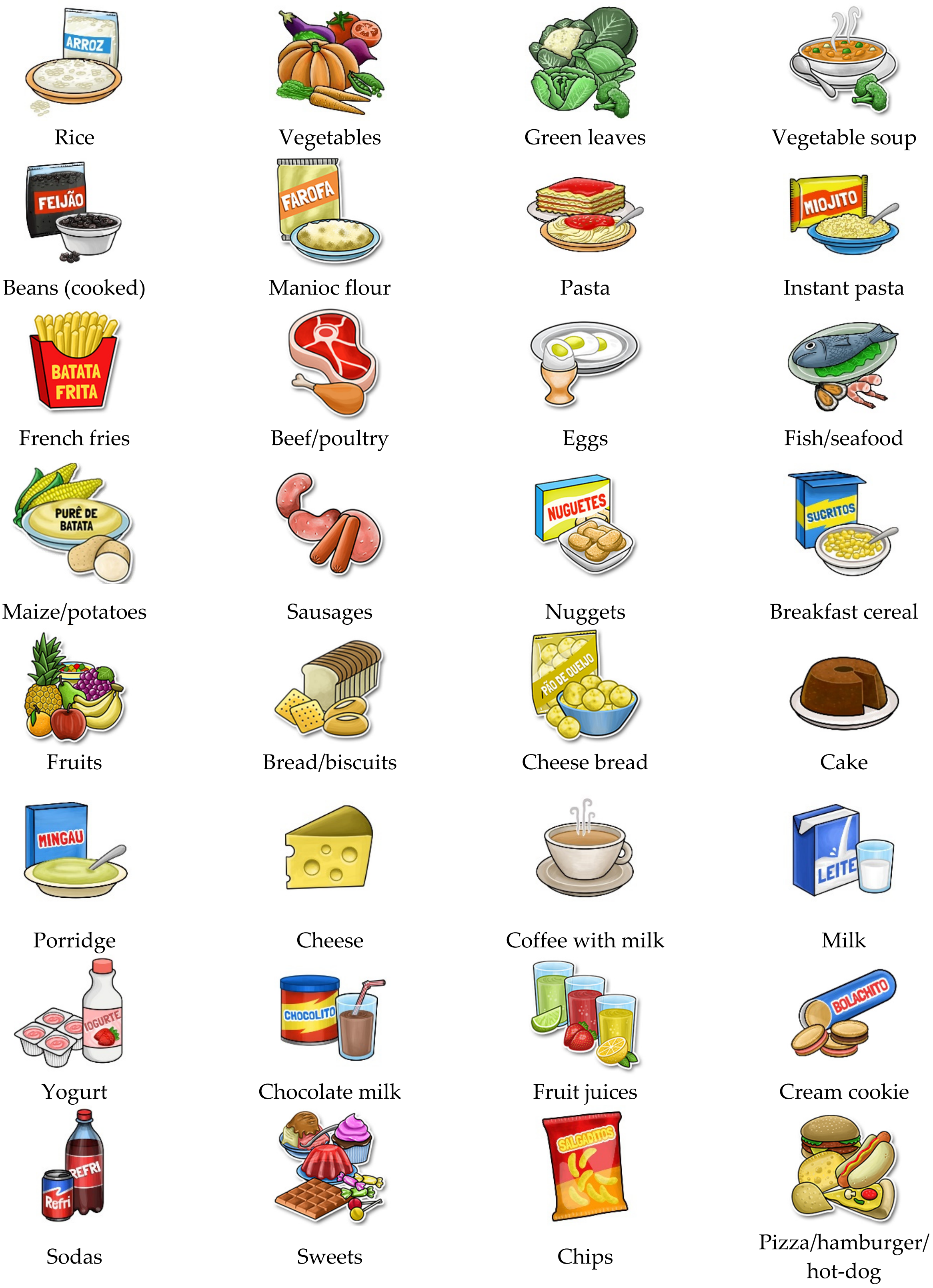

Figure S1. Food and beverage items from WebCAAFE questionnaire, Florianópolis, Southern Brazil, 2015.

Table S1. Intra-group variation values of the five clusters derived from the k-means non-hierarchical cluster analyses, Florianópolis, Southern Brazil, 2015.

	Clusters				
	1	2	3	4	5
**Intra-group values**	28973.60	42564.50	15625.00	54042.83	15512.00

Table S2. Frequencies of conceptual categories by age of 7–10-year-old schoolchildren, Florianópolis, Southern Brazil, 2015.

Age (years)	Total	Evaluative: Preferences	Specific food item name	Food characteristic	Script-scheme	Food preparation	Thematic: combination	Evaluative: health perception	Taxonomic-professional	Nutrient composition	Don't know/ not sure
	n (%)	n (%)	n (%)	n (%)	n (%)	n (%)	n (%)	n (%)	n (%)	n (%)	n (%)
**7 to 8**	57 (42.9)	1 (1.0)	16 (27.4)	3 (5.2)	3 (5.2)	0 (0)	1 (1.0)	3 (5.2)	23 (40.3)	0 (0)	7 (13.5)
**9 to 10**	76 (57.1)	0 (0)	18 (23.7)	3 (4.0)	3 (4.0)	1 (1.3)	1 (1.3)	6 (7.8)	37 (48.8)	1 (1.3)	6 (7.8)

n= absolute frequency; % = relative frequency.
